# Artificial Airway Suctioning: A Systematic Review

**DOI:** 10.7759/cureus.42579

**Published:** 2023-07-27

**Authors:** Nikhil G Sontakke, Mayuri G Sontakke, Naveen K Rai

**Affiliations:** 1 Health Sciences, Jawaharlal Nehru Medical College, Datta Meghe Institute of Higher Education and Research, Wardha, IND; 2 Accident and Trauma Care Technology, Jawaharlal Nehru Medical College, Datta Meghe Institute of Higher Education and Research, Wardha, IND; 3 Biochemistry, Jawaharlal Nehru Medical College, Datta Meghe Institute of Higher Education and Research, Wardha, IND

**Keywords:** oropharyngeal airway suctioning, closed suctioning, gastric lavage, endotracheal suctioning, artificial airway suctioning

## Abstract

Airway suctioning is routinely performed in the majority of care circumstances, including acute care, subacute care, home-based settings, and long-term care. Using an artificial airway to suction the patient allows for the mobilization and evacuation of secretions. When a patient can't independently remove all of the secretions from their respiratory tract, suction is used. This can occur when the body produces excessive secretion or it is not eliminated quickly enough, causing the respiratory system's upper and lower respiratory secretions to accumulate. Airway blockage and inadequate breathing may result from this. Ultimately, this leads to a shortage of oxygen and carbon dioxide from the air, both of which are necessary for ideal cellular activity. Artificial airway suctioning is one of the most crucial components of airway care and a core competency for medical professionals trying to ensure airway patency. Artificial airway suctioning is a standard treatment carried out every day globally and is frequently done in both outpatient and inpatient patients. Therefore, specialists must know the safest and most efficient ways to perform surgery and any potential side effects. In ventilated infants and children, the removal of obstructive secretions by endotracheal suctioning is frequently done. It is unknown how suctioning affects the mechanics of breathing. This study used a prospective observational clinical design to examine the immediate impact of airway resistance in endotracheal suctioning, tidal volume, and dynamic lung regulation in mechanically ventilated adult patients and mechanically ventilated pediatric patients. The preparation, process, and indications for intraoperative fusion treatment in various circumstances are covered in this systematic review.

## Introduction and background

Effective gas exchange depends on managing secretions, especially in patients with an artificial airway. The medical personnel ensures a secure and effective clearance of secretions in patients using artificial airways [1]. Anxiety can cause unwanted sympathetic and parasympathetic system stimulation in patients, which can increase heart rate and blood pressure as well as cause unpleasant, excessive secretions [2]. Along the care continuum, artificial airway suctioning is a routine process carried out every day across the world. Preparing the patient, applying suction using the catheter that has been inserted, and providing postoperative care are all included in this treatment [3]. The principle of artificial airway suctioning using either a tracheostomy tube or an endotracheal tube (ETT) is not without risk, although it is usually regarded as safe. There have been reports of transient adverse effects, including hemodynamic abnormalities, hemorrhage, changes in heart rate, and oxygen desaturation [4]. Poor airway suctioning techniques could result in long-term effects such as airway mucosa damage and hospital-acquired infections [5]. The American Association for Respiratory Care (AARC) published a clinical practice guideline (CPG) in 2010 focused on this procedure [3]. Informed by a thorough literature analysis, 10 recommendations for safe endotracheal suctioning were created by the 2010 AARC CPG for patients with artificial airways and mechanical ventilation. The techniques for artificial airway suctioning have changed throughout time, as has the body of scientific knowledge that underpins modern clinical procedures [1].

## Review

Search methodology

**Figure 1:**
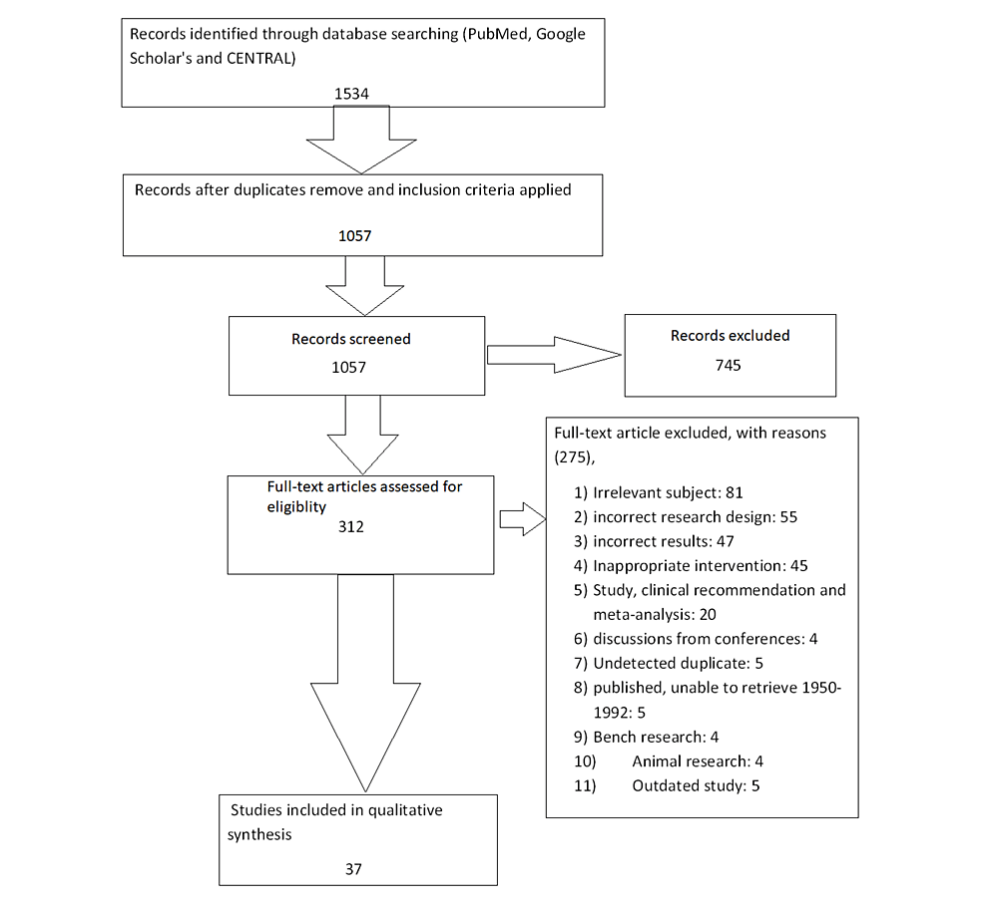
PRISMA flow diagram of search strategy PRISMA: Preferred Reporting Items for Systematic Reviews and Meta-Analyses

We undertook a systematic search through PubMed, Google Scholar, and CENTRAL in December 2022 using keywords such as "artificial airway suctioning", "Endotracheal suctioning", "Gastric lavage", "closed suctioning", and "oropharyngeal airway suctioning"(((artificial airway suctioning[Title/Abstract]) OR ("artificial airway suctioning"[MeSH Terms]), (Endotracheal suctioning[Title/Abstract])) OR (Endotracheal suctioning [MeSH Terms]), (("gastric lavage"[Title/Abstract]) OR ("Gastric lavage[MeSH terms]) AND ("Close suctioning [Title/Abstract]) OR ("close suctioning[MeSH Terms]). One reviewer independently checked the papers that were first retrieved based on title and abstract against the inclusion criteria before moving on to the full texts. Another reviewer also reviewed approximately 20% of these studies to validate inclusion studies. The selection of the studies (Figure 1) depended on the following inclusion criteria: (1) artificial airway suctioning (including endotracheal suctioning and oropharyngeal airway suctioning); (2) suction catheter size for adults and pediatrics; (3) neonatal, pediatric, and adult patients; (4) English language; (5) gastric lavage. The following were the exclusion criteria: (1) case study; (2) natural airway suctioning; (3) animal studies; (4) bench research; (5) not empirical study (e.g., theory or opinion articles), and (6) non-English language research.

Indications of artificial airway suctioning

Six research papers concentrated on the purposes of ETT suctioning [6-11]. One study in neonatal patients discovered an increase in airway resistance (RAW) that was statistically significant (p<0.01), and advised monitoring RAW to figure out if suctioning was necessary [6]. In a study of pediatric patients, ETT suctioning was shown to improve saturation of peripheral oxygen (SpO2) while having no effect on blood pressure, end-tidal carbon dioxide (CO2), or airway pressure in patients exhibiting breathing crackles in the operating theatre and post-anesthesia care unit [7]. Dhakate et al. reported that the nasopharyngeal passage of the ETT appears to be facilitated by a straightforward procedure of nasal cavity dilation with the nasopharyngeal airway (NPA) during induction of anesthesia, which also appears to reduce the incidence and severity of trauma and hemorrhage [8]. Guglielminotti et al. discovered that in adult patients, detectable secretions in the ETT sawtooth pattern on the ventilator flow waveform, and breathing sounds heard while auscultating across the trachea were reliable markers indicating the need for suctioning (p<0.001) [9]. These markers were supported by the results of Sole et al., except for their observable secretions. Although there was a substantial improvement in all of the indicators following suctioning (p<0.001), the amount of secretions (≥0.5mL) collected during suctioning was linked to a few of the previously described symptoms but was not significantly different (p=0.17) [10]. Lucchini et al. noted that there were much fewer suctioning issues when a sound secretion detector was used (p<0.001) and acted as a more accurate indicator of airway secretions, and compared to the signals used in an earlier investigation, mechanical airway suctioning is more necessary [11].

Complications of artificial airway suctioning

Twenty-three research papers looked specifically at the problems caused by mechanical airway suctioning [1,4,12-32]. Seymour et al. found that after minute ventilation, ETT suctioning, quick, shallow breathing index, heart rate, and mean arterial pressure increased significantly. The recovery time ranged between 1 and 7 minutes [12]. Beuret et al. showed that in the adult patient group, suctioning had no discernible influence on the ETT cuff's ability to effectively close the airway [13]. Durand et al. demonstrated that ETT suctioning had a substantial impact on intracranial pressure (ICP), heart rate, perfusion pressure (p<0.05), and mean arterial pressure in neonatal patients [14]. In research by Fanconi, neuromuscular blockers were administered prior to suctioning, and ICP was considerably lower (p<0.001) [15]. Suctioning had no apparent effect on any other physiological reactions. Simbruner et al. noted that while suctioning, mean arterial pressure, heart rate, and transcutaneous partial pressure of oxygen (PO2) all significantly increased, but not at 1, 2, or 5 minutes later [16]. Kaiser et al. found a persistent increase in the velocity of cerebral blood flow following suctioning, which is concerning because this parameter has been linked to brain damage in infants with low birthweight (LBW) [17]. 

Tingay et al. discovered that artificial airway suctioning was linked to significantly lower lung volumes in newborns receiving high-frequency oscillatory ventilation. However, they did see that lung volumes returned to nearly baseline levels within the 60s [18]. Morrow et al. discovered that changes in expired minute ventilation, lung compliance, breathing frequency, and tidal volume were significantly different in the pediatric population after artificial airway suctioning than previously reported [19]. According to Scoble et al., using a suction catheter again after 24 hours had passed had no impact on the prevalence of pneumonia compared to utilizing a fresh catheter for each and every case of artificial airway suctioning [20]. Boothroyd et al. determined that the use of controlled vacuum pressures and graduated suction catheters led to a much lower prevalence of right upper lobe collapse when compared to standard practice [1,21]. When the individual was removed from the ventilator prior to artificial airway suctioning, Fisher et al. discovered that ICP elevations in children with brain injuries were caused by tracheal stimulation from the suctioning process rather than an increase in PCO2 from an apnea [22]. Maggiore et al. discovered that although there were no changes in ventilator days or ICU length of stay (LOS) following the implementation of practice guidelines, adverse events caused by mechanical airway suctioning were considerably reduced [4]. After artificial airway suctioning utilizing both closed and open approaches in an adult population, Jongerden et al. discovered significant increases in mean arterial pressure and heart rate but not SpO2 [23]. Four studies found substantial variations in cerebral perfusion pressure, mean arterial pressure, and ICP after mechanical airway suctioning compared to baseline [1,24-26]. 

Following artificial airway suctioning using closed and open suctioning methods with 100% preoxygenation, Clark et al. found that venous oxygen saturation (SvO2) at 1 and 2 minutes was much reduced, but by 3 minutes, it was back to normal levels [27]. When pre-oxygenated with 100% oxygen for at least 1 minute, Bourgault et al. discovered that artificial airway suctioning using each open and closed approach had no effect on systolic blood pressure or heart rate variability [28]. The Guglielminotti et al. study found that mechanical airway suctioning increased raw and intrinsic positive end-expiratory pressure (PEEP) significantly [29]. Van de Leur et al. discovered that saline solution lavage with significant harm from artificial airway suctioning increases cardiac arrhythmias, oxygen desaturation, blood in respiratory secretions, systolic blood pressure, and subject recollection of the suctioning event when compared to minimally invasive suction with no lavage [30]. Invasive versus minimally invasive artificial airway suctioning techniques did not significantly affect the length of intubation, death, infection, or the amount of time spent in the ICU, according to Van de Leur et al., who also discovered the same negative effects, ignoring subject recollection [31]. Following open ETT suctioning and 100% oxygen hyperinflation, Walsh et al. observed the following changes: cardiac output and functional oxygen saturation (SaO2) were not significantly different, while SvO2 was significantly reduced, and oxygen consumption was significantly increased [32].

Artificial airway suctioning: Routine vs. as-needed only

Lema-Zuluaga et al. found no difference between the two groups in mechanical ventilation days, deaths, ICU LOS, or ventilator-associated pneumonia [33]. There were no studies that examined how often adult patients had to be suctioned. As-needed suctioning is just as beneficial as routine suctioning, and there are no negative consequences for neonatal morbidity or mortality or pediatric populations, according to the evidence provided by these studies. The latest research backs up the 2010 AARC CPG1 recommendation to only use artificial airway suctioning when essential. 

Artificial airway suctioning: Open vs. closed system

Since the early 1990s, when closed suction system devices were developed, the clinical results were compared to those of the conventional open suction system by researchers. Two studies that examined the open and closed systems for managing secretions did so. Adult patients underwent intubation in Witmer et al.'s study, where researchers looked at the volume of secretions suctioned out of artificial airways and discovered that the amount removed using an open suction system (median 1.9 g) and a closed suction system (median 1.7 g) did not statistically differ from one another [34]. In adult patients who were intubated, Lasocki et al. examined how the open and closed suction systems affected the evacuation of secretions. They found that the open suction system's mean aspirate mass (mean 3.2 g) was higher than the closed suction system's mean aspirate mass (mean 0.6 g) [35]. Numerous studies have assessed how the open suction system and the closed suction system affect physiologic parameters. Adult patients were utilized in studies that examined variables such as breathing frequency, SpO2, ICP, cardiac dysrhythmias, agitation, heart rate, and pain. Although some studies did find some slight changes, the majority of these studies revealed no significant differences in the results between the closed and open suction systems. Johnson et al. observed that closed suction systems exhibited fewer physiologic disturbances (blood pressure, SpO2, heart rate, and dysrhythmias) compared to open suction systems [36]. According to Lasocki et al., the closed suction system maintained better oxygenation and ventilation [35]. An adult patient with an artificial airway can have secretions removed safely and effectively using either the closed suction system or the open suction system, depending on the quantity and quality of the supporting evidence [1].

Artificial airway suctioning: Clean vs. sterile

Secretions from an artificial airway can be safely and successfully removed using both the open and closed suction systems. A safe environment must be maintained for the patient and the clinician when executing an open artificial airway suctioning treatment to ensure protection from clinical pathogens. However, concerns are raised about whether the technique should be sterile, i.e., devoid of germs or other microbes, or clean, which is free of visible visual contamination. Some medical professionals contend that the sterility of the physician is unimportant because a non-sterile field will be crossed by the open suction catheter, making subsequent passes non-sterile [37]. Tracheal suctioning increases the chance of cross-contamination; however, using an aseptic (sterile) approach can reduce the chance of cross-contamination. Regarding the differences in clinically important outcomes between the clean and sterile techniques for artificial airway suctioning, there is a gap in the literature. To advocate for more studies to close this knowledge gap may be unethical given the definite possibility of injury from a less-than-sterile environment. During open suctioning events, the patient must be protected from any cross-contamination; it is advised that the doctor employ sterile techniques whenever possible [1].

Normal saline solution lavage for artificial airway suctioning

The consequences of utilizing ordinary saline solution during artificial airway suctioning have been the subject of extensive research [1]. Theoretically, increasing the volume of secretions and liquifying secretions extracted during suctioning are benefits of utilizing normal saline solution [2]. Nine studies that examined the effects of using a regular saline solution during artificial airway suctioning on oxygenation were found after a study of the literature [1,38-45]. The findings of seven of these investigations imply that oxygenation may be adversely affected by ordinary saline solution. These investigations, however limited, suggest that artificial airway suctioning might have a negative effect on measures including PO2, SpO2, and SvO2 [38,40-45]. Even while other physiological measurements like blood pressure and heart rate are unaffected [1,39]. Using a regular saline solution while suctioning an artificial airway has also been demonstrated to make people under the age of 60 more dyspneic [1]. A randomized trial was conducted by McKinley et al. to assess the duration of invasive mechanical breathing and intubation in pediatric patients. They discovered that using neither a saline solution nor none at all was equally effective [46].

Artificial airway suctioning vs. bronchoscopy

In a study by Qiao et al., 73 randomly chosen individuals were treated for acute chronic obstructive pulmonary disease (COPD) episodes. They were split into two groups receiving routine artificial airway suctioning: one group received conventional artificial airway suctioning alone, and the other received conventional artificial airway suctioning with the addition of bronchoscopy. According to their research [47], the outcomes for mortality, number of days using mechanical ventilation, successful feeding, and days of invasive mechanical ventilation were significantly improved when routine bronchoscopy was combined with conventional artificial airway suctioning. 

Applied vacuum pressure and suction catheter size

There is a lack of studies examining the security and efficiency of various vacuum pressures and catheter diameters [1]. Javadi et al. conducted a within-subjects repeated-measures study to assess the effects of various suction catheter sizes on adult subjects' blood pressure, heart rate, embedded Image, secretion volume, and discomfort. According to the study procedure, all of the participants in their study underwent intubation using an ETT with a size 7.5 inner diameter, and they were all suctioned using either 12 French or 14 French suction catheters. Both sizes of catheters were used to suction all of the participants. With the use of the bigger catheter, they observed a considerable rise in systolic blood pressure, discomfort, secretion volume, and heart rate [48]. The impact of suction catheter vacuum pressures (80, 100, and 120 mm Hg) and outer diameter size in relation to ETT inner diameter size (small: 0.4; medium: 0.7; and large: 0.9) on several physiologic indices in pediatric participants was evaluated in prospective research by Singh et al., where they discovered that the physiologic parameters were affected identically by all suction catheter sizes at different pressures [49]. The previous norm did not make a formal recommendation about applied vacuum pressure since there was not enough data to do so. There is little evidence to support maintaining suction pressures below -200 mm Hg in adults and between -80 and -100 mm Hg in newborns [1]. A summary of all the articles included in this review is listed in Table 1.

**Table 1:** Summary of the articles included in the review ETCO2: End-tidal carbon dioxide, NPA: Nasopharyngeal airway, ETT: Endotracheal tube, TBA: Total bile acid, PVC: Polyvinyl chloride, ICP: Intracranial pressure, CPP: Cerebral perfusion pressure, PO2: Partial pressure of oxygen, TcPO2: Transcutaneous oxygen pressure, CBFv: Cerebral blood flow velocity, VLBW: Very low birthweight, HFOV: High-frequency oscillatory ventilation, RUL: Right upper lobe, PCO2: Partial pressure of carbon dioxide, MAP: Mean airway pressure, SpO2: Oxygen saturation, CSS: Closed suction system, OSS: Open suction system, SvO2: Venous oxygen saturation, HR: Heart rate, SBP: Systolic blood pressure, TS: Tracheal suctioning, ETS: Endotracheal suctioning, SaO2: Arterial oxygen saturation, CO: Carbon monoxide, VO2: Volume of oxygen, AECOPD: Acute exacerbations of chronic obstructive pulmonary disease

Authors	Year	Country	Findings
Blakeman et al., [1]	2022	USA	Artificial airway suctioning is crucial for proper management, but risks and complications must be considered.
Maggiore et al., [4]	2013	Italy	Endotracheal suctioning can cause adverse effects; guidelines can reduce incidence.
Prendiville et al., [6]	1986	England	Little difference in respiratory system resistance between babies with and without hyaline membrane disease on the first or second days.
Seyedhejazi et al., [7]	2019	Iran	Study shows open and deep suction improves oxygen saturation, reduces complications, and emergence time; no positive effect on airway pressure, ETCO2, blood pressure, and respiratory rate.
Dhakate et al., [8]	2020	India	NPA nasal cavity dilation eases ETT insertion, reduces trauma, and bleeding during intubation.
Guglielminotti et al., [9]	2000	France	Sawtooth pattern and/or respiratory sounds indicate retained secretions in patients; absence may rule them out.
Sole et al., [10]	2015	USA	Patients receiving mechanical ventilation should be routinely assessed for coarse crackles over the trachea, the most common indicator for endotracheal suctioning.
Lucchini et al., [11]	2011	Italy	TBA care provides accurate suctioning indications, anticipates deterioration, and reduces unnecessary suctioning.
Seymour et al., [12]	2009	USA	Significant cardiac and respiratory changes in spontaneously breathing mechanically ventilated patients after closed-system suctioning, lasting longer than in sedated patients.
Beuret et al., [13]	2012	France	Study shows single tracheal suctioning maneuver doesn't increase aspiration risk in PVC-cuffed tracheal tube cuff patients under certain conditions.
Durand et al., [14]	1989	USA	ETT suctioning significantly increases BP, ICP, and CPP in preterm infants on assisted ventilation, independent of oxygenation and ventilation changes.
Fanconi et al., [15]	1987	Switzerland	Suctioning reduced heart rate and transcutaneous Po2, but pancuronium did not alter these changes, and no statistical difference was observed.
Simbruner et al., [16]	1981	Austria	During tracheal suction, the TcPo2 and heart rate decreased but the blood pressure increased. Bag ventilation with pure oxygen reversed all these changes.
Kaiser et al., [17]	2008	USA	Prolonged CBFv increases in ventilated VLBW infants; concerning as disturbances may cause brain injury.
Tingay et al., [18]	2007	Australia	ETT suction causes temporary lung volume loss in newborns with HFOV.
Morrow et al., [19]	2006	South Africa	Study shows endotracheal suctioning causes a drop in dynamic compliance and expired tidal volume in ventilated children with lung pathology.
Scoble et al., [20]	2001	Australia	Pneumonia in control group and study group differed by 0.7%, resulting in cost savings of $4.14 per patient per day.
Boothroyd et al., [21]	1996	England	High negative pressure and deep-suctioning cause RUL collapse in children, prolonging intensive care and potentially causing morbidity.
Fisher et al., [22]	1982	USA	Examining tracheal stimulation and PCO_2_ changes in ICP during endotracheal suctioning to prevent neurologic deterioration.
Jongerden et al., [23]	2012	Netherland	Quantified changes in heart rate, MAP, and Spo_2_ in patients undergoing endotracheal suctioning with CSS and OSS.
Kerr et al., [24]	1999	USA	Increased jugular venous oxygen tension, middle cerebral artery velocity, and arterial pressure indicate maintained cerebral oxygen delivery.
Gemma et al., [25]	2002	Italy	Sedation level should be deepened for head injury patients coughing or moving during endotracheal suctioning.
Brucia et al., [26]	1996	USA	Research suggests minimizing airway stimulation during the suctioning procedure.
Clark et al., [27]	1990	USA	Closed suction method improves SvO2 after endotracheal suctioning, requiring hyperoxygenation.
Bourgault et al., [28]	2006	USA	ETT suctioning increased HR, SBP, and PO2 without significant effects on autonomic HR modulation mechanisms.
Guglielminotti et al., [29]	1998	France	TS evokes a temporary bronchoconstrictor response, but respiratory resistance remains unreduced, and effective beta2-adrenergic blockade fails.
Van de Leur et al., [30]	2003	Netherland	Minimally invasive airway suctioning reduces recollection but doesn't cause discomfort.
Van de Leur et al., [31]	2003	Netherland	Study shows minimally invasive airway suctioning in intubated ICU patients has fewer side effects, shorter intubation, longer stay, and lower mortality.
Walsh et al., [32]	1989	USA	ETS significantly decreased SvO_2_ due to increased VO_2_ and CO, while SaO_2_ changes were modest and insensitive.
Lema-Zuluaga et al., [33]	2018	Colombia	Hypoxemia occurs when the saturation drops by 10% during or after ET suctioning, while accident extubation events and cardiopulmonary arrest occur. Arrhythmias, such as bradycardia or tachycardia, are present during or after the suction.
Witmer et al., [34]	1991	USA	No significant difference in secretions removed from artificial airways using open or closed suction systems.
Lasocki et al., [35]	2006	France	Closed circuit endotracheal suctioning prevents hypoxemia, increases suctioning pressure, and enhances gas exchange efficiency.
Ackerman et al., [39]	1998	USA	Normal saline before suctioning negatively impacts oxygen saturation in patients with pulmonary infection.
McKinley et al., [46]	2018	Australia	No saline was as effective as 0.225% or 0.9% saline in endotracheal suctioning; optimal policy: routine use, occasional 0.9% saline for thick secretions.
Qiao et al., [47]	2018	China	Bronchoscopic sputum suction and mechanical ventilation effectively treat AECOPD patients.
Javadi et al., [48]	2017	Iran	Use a smaller catheter for suction to reduce heart rate and blood pressure, and carefully select the catheter diameter to prevent prolonged arterial oxygen saturation loss.
Singh et al., [49]	1991	England	Oral suctioning can cause physiological changes; follow precautions like tracheal suctioning.

## Conclusions

An essential part of managing artificial airways is suctioning. Depending on the patient's clinical state, blood oxygen saturation, pace of breathing, and visibility of airway secretions, it is carried out as necessary. The process aids in enhancing lung airflow. Artificial airway suctioning is used in various cases, such as when the patient can't remove all secretions by themselves. The excessive amount of secretion, if not removed quickly, can lead to airway blockage and inadequate breathing, resulting in a shortage of oxygen. Therefore, artificial airway suctioning is a common treatment that is carried out every day all over the globe and is frequently done in both inpatient and outpatient settings. All artificial suctioning methods (such as routine, close, open, sterile, and clean) are beneficial for removing secretions from the mouth or respiratory tract. There are no negative consequences of artificial airway suctioning on adult morbidity or mortality in adult populations.
